# Antibacterial and Wound-Healing Activities of Statistically Optimized Nitrofurazone- and Lidocaine-Loaded Silica Microspheres by the Box–Behnken Design

**DOI:** 10.3390/molecules27082532

**Published:** 2022-04-14

**Authors:** Hafeez Ullah Khan, Fahmeed Nasir, Safirah Maheen, Syed Salman Shafqat, Shahid Shah, Ahmed Khames, Mohammed M. Ghoneim, Ghulam Abbas, Saleha Shabbir, Mohamed A. Abdelgawad, Mohammad A. S. Abourehab, Amna Irfan, Amani M. El Sisi

**Affiliations:** 1 Department of Pharmaceutics, College of Pharmacy, University of Sargodha, Sargodha 40100, Pakistan; hafeezullah.khan@uos.edu.pk (H.U.K.); fameechrist1@yahoo.com (F.N.); salehashabbir909@gmail.com (S.S.); amnairfan300@gmail.com (A.I.); 2 Department of Chemistry, University of Education, Lahore 54000, Pakistan; salman.shafqat@ue.edu.pk; 3 Department of Pharmacy Practice, Faculty of Pharmaceutical Sciences, Government College University Faisalabad, Faisalabad 38000, Pakistan; shahid.shah@gcuf.edu.pk; 4 Department of Pharmaceutics and Industrial Pharmacy, College of Pharmacy, Taif University, Taif 21944, Saudi Arabia; a.khamies@tu.edu.sa; 5 Department of Pharmacy Practice, Faculty of Pharmacy, AlMaarefa University, Ad Diriyah 13713, Saudi Arabia; mghoneim@mcst.edu.sa; 6 Department of Pharmaceutics, Faculty of Pharmaceutical Sciences, Government College University Faisalabad, Faisalabad 38000, Pakistan; 7 Department of Pharmaceutical Chemistry, College of Pharmacy, Jouf University, Sakaka 72341, Saudi Arabia; mhmdgwd@ju.edu.sa; 8 Department of Pharmaceutics, Faculty of Pharmacy, Umm Al-Qura University, Makkah 21955, Saudi Arabia; maabourehab@uqu.edu.sa; 9 Department of Pharmaceutics and Industrial Pharmacy, Faculty of Pharmacy, Beni-Suef University, Beni-Suef 62514, Egypt; amany.elsese@pharma.bsu.edu.eg

**Keywords:** antibacterial therapeutics, Box–Behnken design, lidocaine, nitrofurazone, silica microspheres

## Abstract

In the current study, nitrofurazone- (NFZ) and lidocaine-loaded (LD) silica microspheres were fabricated to address pathological indications of skin infections. The microspheres were prepared by the sol–gel method applying the Box–Behnken design and evaluated for size distribution, morphology, zeta potential, physico-chemical compatibility, XRD, thermogravimetric analysis, antibacterial and cytotoxicity activities. The comparative in vitro drug release study of microspheres revealed a 30% release of NFZ and 33% of LD after 8 h. The microspheres showed 81% percentage yield (PY) and 71.9% entrapment efficiency. XRD patterns confirmed the entrapment of NFZ–LD in silica microspheres with a significant reduction in crystallinity of the drugs. Thermal and FTIR studies proved the absence of any profound interactions of the formulation ingredients. The smooth spherical microspheres had a −28 mV zeta potential and a 10–100 µm size distribution. In vitro antibacterial activities of the NFZ–LD microspheres showed an increased zone of inhibition compared to pure drug suspensions. The in vivo efficacy tested on rabbits showed a comparatively rapid wound healing with complete lack of skin irritation impact. The cytotoxicity studies revealed more acceptability of silica microspheres with negligible harm to cells. The study suggests that the NFZ- and LD-loaded silica microspheres would be an ideal system for accelerating and promoting rapid healing of various acute and chronic wounds.

## 1. Introduction

Controlled release drug delivery systems are designed to provide medications over an extended period of time with minimal side effects. Such systems can deliver synthetic, semisynthetic, and biological substances with equal efficiency. Controlled-release formulations produced by sol–gel techniques exhibit an excellent tissue response without creating any wound inflammation [1]. The sol–gel technique uses silica [SiO_2_], a biodegradable, biocompatible, and exudated adsorbent carrier which can be produced via acid hydrolysis of tetraethyl orthosilicate (TEOs). Several silica-based sol–gel formulations have been reported in the literature for both biomedical and controlled-release applications [2,3]. The lack of moisture in standard wound dressings usually hinders wound healing by producing and increasing bacterial growth [4]. To avoid irritation, greasiness, erythema, burning and pruritic rashes [5], topical formulations are not allowed on wounds for long durations. Traditional drug delivery systems produce a number of local adverse effects, such as a burning sensation, skin irritation, greasiness, stinging, pruritic rash, erythema, and tenderness, which make them less acceptable. The proposed silica microspheres are ranked higher because they can overcome all of the mentioned problems associated with the conventional mode of treatment. Regarding the application of DLMs, there is no issue of viscosity as observed with conventional systems, and such microspheres showed comparatively longer duration of action with better penetration of drugs through dermis without repetitive use [6]. Moreover, hydrolysis, aggregation, and premature drug leakage are the disadvantages associated with novel drug carriers such as hydrogels, liposomes, and niosomes. Phospholipids, a key component of liposomes, are also more prone to oxidation–reduction processes, which further destabilizes liposomes [7]. In contrast, the, sol–gel technique is used for preparing silica microspheres because it provides the optimum drug release kinetics [3]. Different studies found that silica-based microparticles lasted longer and penetrated deeper into the dermis than conventional medicines, promoting faster wound healing [7,8]. Moreover, silica has been shown to improve re-epithelialization, epidermal maturity and revascularization in diverse wounds including burn wounds [9]. It also prevents bacterial growth at wound sites without causing inflammation [10].

Despite proper diagnosis and antibiotic therapy for infected or burn wounds, substantial microbial resistance and side effects may still occur. A unique medication delivery system concentrating on regulated, sustained, and prolonged administration of a therapeutic agent may appear successful [11]. In the current study, NFZ and LD were encapsulated in silica microspheres [12] and used on highly resistant second- and third-degree potential burn infections, skin infections pyodermas, wounds, infected dermatoses and skin grafting instances where highly resistant bacterial growth may cause graft rejection or donor infection [13]. The NFZ and LD worked well against both Gram-positive and Gram-negative bacteria and especially well against sulfonamide-resistant bacteria. However, its frequency of daily application is very high due to its poor penetration and its frequent use might result in erythema, pruritus, itching, edema, rashes, inflammation and dermatitis which decreased patients’ compliance. Moreover, in all the pathological conditions where NFZ is needed, it also requires the use of an equally effective analgesic agent. Local anesthetics such as LD can be utilized for this purpose. However, by increasing cell permeability, it induces the rupture of cell membranes and the release of important intracellular components. An improved healing impact, lower germ resistance, and synergistic therapeutic response with reduced toxicity may be substantial benefits of such a combination [14]. Because of their high biocompatibility, combination therapy loaded silica microspheres appear to be a potential medication delivery strategy for treating various skin infections [15,16].

The current study investigated the use of silica microspheres to provide LD and NFZ combination therapy to enhance rapid wound healing. The pH (*X*_1_), stirring time (*X*_2_), and sunflower oil concentration (*X*_3_) were studied numerically for their impact on gelation time (GT) of formulations (Y_1_), PY (Y_2_), percentage release of NFZ (Y_3_) and LD (Y_4_). A measurement of 100 mL sunflower oil was used for the emulsification process of the sol–gel technology. When the sunflower oil concentration and the stirring time were increased, it resulted in an increase in the PY and entrapment efficiency of the drugs. Before applying the Box–Behnken design, a set of preliminary trials were conducted to measure the level of formulation variables. The range of level of variables which give some desirable results of GT, PY and release of NFZ and LD, was then further elucidated by using the design. Formulation component compatibility was evaluated using FT-IR, XRD and thermal gravimetric analyses (TGA). The selected optimized NFZ- and LD-loaded microspheres were further evaluated for in vitro and in vivo antibacterial efficacy, skin irritation and cytotoxicity studies.

## 2. Results and Discussion

### 2.1. Optimizing Variables of Drug-Loaded Microspheres (DLMs)

It has been observed that the gelation time (GT) of preparation F14 was 8 min at pH 5.83, while F7 and F9 had shown 76 min at pH 7.1 and 149 min at high pH 8.4. It has been observed that the GT increased with the increase in pH due to decreased activity of catalyst 0.1 M HCl and the increased concentration of 0.08 M NH_4_OH. When the concentration of the base was increased, the effect of the catalyst became low, resulting in a slow hydrolysis reaction. Ultimately, the GT increased. Gelation is related to the hydrolysis of ethyl silicate, and the study established that at low pH, the reaction proceeded faster which resulted in less GT and vice versa [1,17]. The polynomial equation generated by the Box-Behnken design for the study of interactions of independent variables on GT is given below:

(1) $$\begin{array}{c} \mathrm{GT}\;=+75.95+69.50X_{1}+0.1539X_{2}-1.85X_{3}-2.31X_{1}X_{2}+0.6923X_{1}X_{3}\\ +1.01X_{2}X_{3}+0.9935X_{1}^{2}+0.3025X_{2}^{2}-0.6975X_{3}^{2} \end{array}$$

It has been observed from the values of the polynomial equation that *X*_1_ (69.50) has a significant effect on GT (Y_1_), and the interaction terms *X*_1_*X*_3_ and *X*_2_*X*_3_ showed a significant synergistic interaction effect on Y_1_ because they showed a positive sign. The higher level of all studied variables did not produce any significant impact except on the pH level for which $X_{1}^{2}$ (0.9935) showed a synergistic effect on the response of Y_1_. PY has been observed in the range of 34% to 84% for 17 formulations of DLMs. The maximum yield of 84% was obtained for F13 at a stirring time of 3 h and a vegetable oil concentration of 100 mL. The formulation F6 showed an average PY of 61% at a stirring time of 2 h and a vegetable oil level of 75 mL, while F15 showed a minimum PY of 34% because it was processed for a stirring time of 1 h and vegetable oil of 50 mL depicting a prominent influence of stirring time and vegetable oil on the variation of PY. Sufficient stirring time provides a suitable time interval for a reaction to produce microspheres, while vegetable oil concentration provides a medium for the precipitation of microspheres. That is why when the stirring time and vegetable oil concentration increased, the PY also increased and vice versa. The formulation F17 showed a PY of 64% at a stirring time of 3 h which could be associated with a medium concentration of oil (75 mL). Hence, it is established that both independent variables, namely stirring time and vegetable oil concentration, have prominent effects on PY [18].

(2) $$\mathrm{PY}\;=+58.34+0.5754X_{1}+8.75X_{2}+14.75X_{3}-1.00X_{1}X_{2}-0.0046X_{1}X_{3}+\;1.60X_{2}X_{3}-1.47X_{1}^{2}+4.382X_{2}^{2}+2.62X_{3}^{2}$$

**Figure 1:**
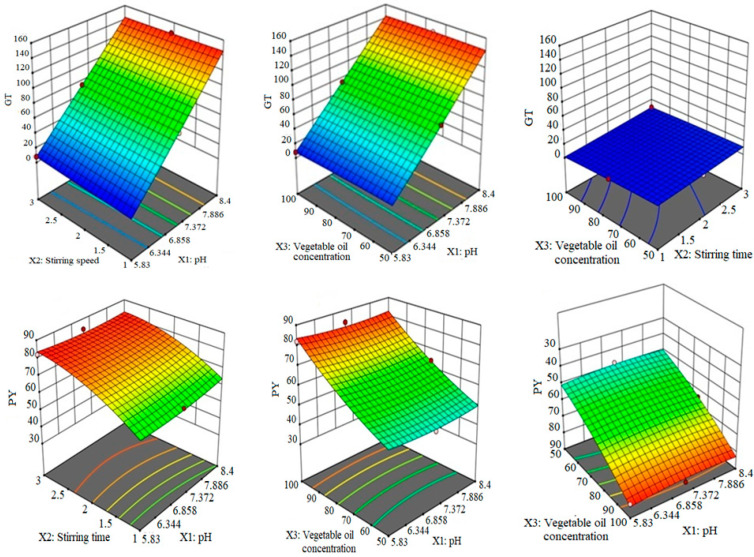
The 3D surface plots indicating the cumulative impact of stirring time, pH, and vegetable oil concentration on GT and PY.

The polynomial equation for PY shows that $X_{3}$ (14.75) and $X_{2}$ (8.75) had produced a significant effect on PY. Similarly, the interaction of *X*_1_ with both *X*_2_ and *X*_3_ showed prominent antagonistic effects, while the interaction term $X_{2}X_{3}$ (1.60) showed a synergistic impact on PY. The effect of a higher concentration of stirring time and oil concentration was also observed to be positive for PY. The diagrammatic presentation of the impact of formulation variables on GT and PY is depicted in Figure 1.

The in vitro release of NFZ- and LD-loaded silica microspheres was performed in a basic media buffer (pH 6.8). The formulation F14 formulated at a lower pH 5.83 showed a release of 30% and 33.3% for NFZ and LD, respectively, whereas F17 formulated at pH 8.4 showed a fast release of 43.8% of NFZ and 45.09% of LD (Table 1). The formulation F4 formulated at pH 7.1 exhibited a moderate release of 36.8% of NFZ and 37.9% of LD. This slow, high and moderate drug release from silica microspheres was because of the formulation pH at which they were synthesized. The presence of a low pore volume of the silica formulations was achieved at the low pH of the sol [2]. Hence, the drug release was slow. In the same way, high pH resulted in high pore volume and faster drug release. The effect of larger pore volume was that a larger amount of water penetrated into the pores, and diffusional release of drugs increased. The release of drugs from microspheres was observed to be biphasic, characterized by the fast-initial release then slow release. Similar findings have been reported in the literature [3]. Moreover, the release of LD was found to be faster than the release of NFZ which could be associated with the higher water solubility of LD compared to NFZ. The polynomial equations generated in the Box–Behnken design for drug release are given below:

(3) $$\mathrm{NFZ}\;\mathrm{Release}=+35.41+6.22X_{1}-0.1734X_{2}-0.0068X_{3}+0.2968X_{1}X_{2}\;+0.7635X_{1}X_{3}+0.0399X_{2}X_{3}+1.05X_{1}^{2}+0.9844X_{2}^{2}\;+0.2177X_{3}^{2}$$

(4) $$\mathrm{LD}\;\mathrm{HCl}\;\mathrm{Releaas}=+36.49+4.33X_{1}-1.33X_{2}+0.3672X_{3}+0.309X_{1}X_{2}\;+0.1907X_{1}X_{3}-0.1567X_{2}X_{3}+2.59X_{1}^{2}+2.54X_{2}^{2}-0.2177X_{3}^{2}$$

**Figure 2:**
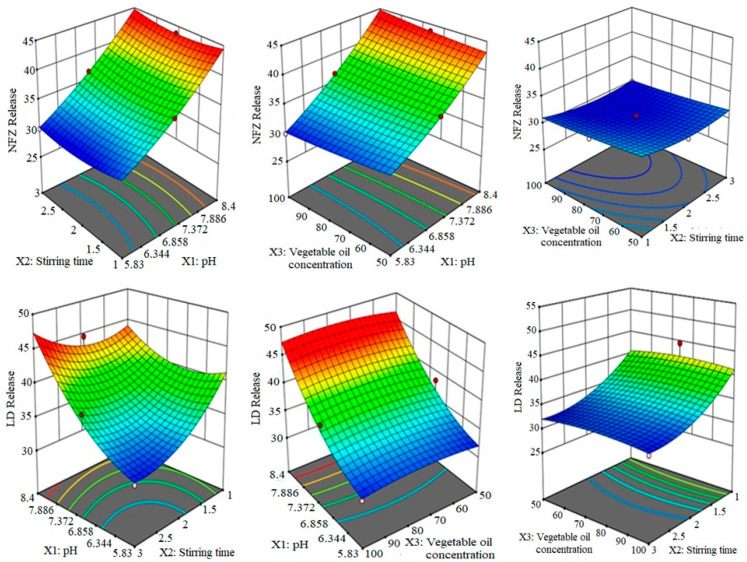
**The** 3D surface plots indicating the cumulative impact of stirring time, pH and vegetable oil concentration on the in vitro release of NFZ and LD.

It has been quite evident that $X_{1}$ (6.22) has a significant effect on the NFZ release (Y_3_) and the LD release (Y_4_) because its coefficient value is higher than other variables. As a result, the adjustment of the formulation pH could be considered as a key and critical factor in controlling drug release from silica DLMs. Both $X_{1}X_{2}$ and $X_{1}X_{3}$ clearly showed synergistic interactive impact on the release of both drugs. The statistical analysis of variance (ANOVA) for the individualized and cumulative effect of the formulation factors on the studied responses is shown in Table 1. The equations depicted that higher levels of variables brought a significant synergistic impact on release profiles. The diagrammatic presentation of the impact of formulation variables on the release of NFZ and LD is depicted in Figure 2. To evaluate the drug release mechanism, five different kinetic models were applied on the release data by using DD solver software, and the best matched release model was selected on the basis of the correlation coefficient (R^2^) of the applied model. Through comparison, it was confirmed that the release of NFZ and LD from microspheres followed the Higuchi model [19]. The Higuchi equation has commonly been applied for the diffusion-controlled release from a homogeneous matrix or from a porous matrix from which a drug is leached by the bathing fluid that penetrates the matrix through pores and capillaries [20]. The Korsmeyer–Peppas equation ‘*n*’ values obtained for DLMs suggested that the NFZ followed non-fickian transport (0.559) because the value of “*n*” lies between 0.45 and 0.89, while LD followed super IIA transport (1425) because the value of “*n*” is greater than 0.89. This behavior is attributed to the porous nature of silica-based microspheres [21].

**Figure 3:**
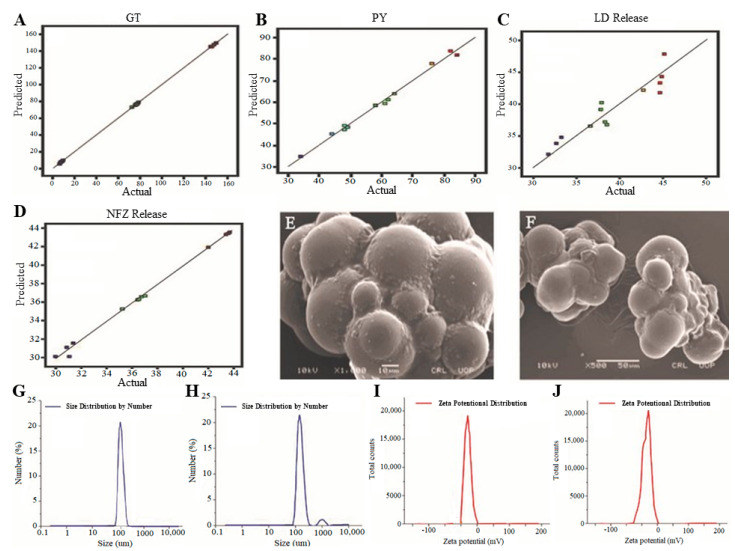
Comparative graphs of predicted versus experimental results of GT (**A**); PY (**B**); LD release (**C**); NFZ release (**D**) SEM microphotographs (**E**,**F**); zeta size analysis curve (**G**,**H**); and zeta potential curve (**I**,**J**); of drug-unloaded and drug-loaded optimized silica microspheres.

The quadratic model showed significant F-values for all studied variables, and adequate precisian was observed to be significant for Y_1_ (357.0157), Y_2_ (37.4556), Y_3_ (43.3943) and Y_4_ (9.4454). It has been shown that the predicted R-squared values of all variables were in reasonable agreement with adjusted R-squared values, suggesting the adequacy of the applied model design. The predicted values of all studied responses (Figure 3) were found to be closely matched with their experimental values that indicated that the DLMs were found to be quite reasonable and reliable for the studied responses [22]. To further validate the experimental model, the optimized batch of silica microspheres (Table 2) was prepared using suggested optimal levels of independent variables by software Design Expert version-11.

### 2.2. Scanning Electron Microscope Analysis for DLMs

Surface morphology of the optimized drug-loaded microspheres was also examined at different magnifications by SEM. The SEM images showed that drug-loaded microspheres have spherical and smooth surfaces. Moreover, SEM analysis revealed the absence of clumps and the agglomeration of microspheres as shown in Figure 3. The absence of clumps or agglomeration is an indication toward higher stability and better release properties of microspheres [1].

### 2.3. Zeta Potential Analysis

Zeta potential is used to calculate the surface charge which can be further used to evaluate the stability of developed formulations [22]. The zeta potential analysis of the optimized DLMs displayed only one peak at −28 mV which covered a 100% area (Figure 3). This indicated the absence of any positive charge as shown in Figure 3. In this way, there is no possibility of attracting opposite charges, and DLMs showed higher stability. This completely negative charge of microspheres is due to the presence of silica (SiO_2_) because silica contains a negative charge. Microspheres have been found to be suitably stable because the value has been observed in the range of ±20–30 mV which indicates stability of the formulation [23].

### 2.4. Size Distribution by Zeta Sizer

Size distribution analysis was performed for the optimized DLMs. The optimized formulation showed the size range from 94.5 μm to 487.5 μm as shown in Figure 3. The average size was 253 μm. As shown in Figure 3, 94.7% particles have a size range of 94.5 μm to 487.5 μm, and only 5.3% of the microparticles have 915 μm to 1176 μm. This reduced size is due to the high stirring speed. The increase in stirring speed brought a reduction in the size of the microparticles. The particle and zeta potential with the polydispersity index (PDI) of all 17 formulations is given in Supplementary Material Table S2 of the supplementary section. The PDI of DLMs was 0.607. It is established that a PDI value of 0.1 to 0.25 shows a narrow size distribution and a PDI value more than 0.5 indicates a very broad distribution [24].

### 2.5. Fourier Transforms Infrared Spectrophotometric Analysis

**Figure 4:**
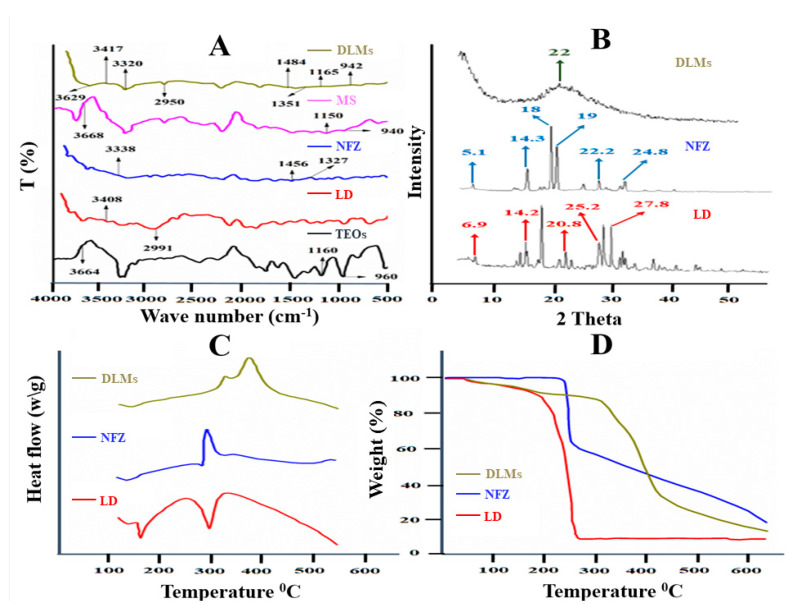
FTIR patterns (**A**); XRD spectra (**B**); thermograms (DSC & TGA) of drug-loaded-optimized silica microspheres and their formulation components (**C**,**D**).

The FTIR analysis was conducted to evaluate possible interactions between NFZ and LD when loaded in silica microspheres. The FTIR analysis was performed for ethyl silicate, NFZ, LD, silica microspheres and drug-loaded microspheres as presented in Figure 4. Ethyl silicate showed absorption at the wave number of 1160 cm^−1^, corresponding to Si–O–Si stretching [25,26]. An absorption band appeared at 960 cm^−1^ linked to the stretching frequency of Si–O–H. The OH group showed absorption at the wave number of 3664 cm^−1^ stretching. Silica microspheres exhibited similar peaks to ethyl silicate with just a slight shift in peaks. LD showed absorption wave values at 2991 cm^−1^ which corresponds to C-H stretching, whereas N–H stretching showed a value at 3408 cm^−1^ [3]. NFZ showed a wave value at 1327 cm^−1^ stretching, which corresponds to the C–N group, whereas the absorption value was observed at 1456 cm^−1^ stretching, corresponding to the N–O group. The N–H group showed a stretching value at the wave number of 3338 cm^−1^ as shown in Figure 4. The FTIR analysis of drug-loaded microspheres showed all major peaks of ethyl silicate, NFZ and LD which indicates the absence of strong interactions between them [27].

### 2.6. Thermal Analysis

DSC was performed to evaluate the degree of crystallinity of NFZ and LD in the optimized formulation. The differential scanning calorimetric (DSC) curve of LD showed only two profound endothermic peaks at 95.82 °C and at 269.42 °C as shown in Figure 4. The first endothermic peak was near its melting point [27]. The DSC curve of NFZ exhibited only one sharp exothermic peak at 263.74 °C which was related to the decomposition of the drug due to pyrolysis (Figure 4). The DSC thermograms of the drug-loaded silica microspheres presented two distinct exothermic peaks at 309.62 °C and 369.46 °C, respectively. These two exothermic peaks correspond to the decomposition of the microspheres, and to the second profound exothermic peak which was at 309.62 °C and corresponds to the almost complete decomposition of DLMs due to pyrolysis. The absence of peaks close to the melting points of NFZ and LD confirm that drugs are present in an amorphous form in the formulation [28].

The thermogravimetric curve for LD exhibited weight loss at two points. The first weight loss was at 213.93 °C with the remaining sample at 89.02% due to the loss of moisture. The second major weight loss of 90.68% at 272.93 °C was linked to the degradation process. In the TGA analysis of NFZ, the first weight loss of 1.46% was observed at 247.34 °C which was attributed to the removal of moisture. Another weight loss (37.29%) observed at 265.82 °C was due to degradation. The TGA analysis of DLMs displayed three phases of weight loss. Initial weight loss of 2.81% was observed at 135.7 °C followed by a second weight loss of 6.74% at 284.31 °C. The third weight loss was found at 391.66 °C and the remaining sample was 67.17%. The first weight loss, which was found to be abrupt, corresponds to the removal of water and some other impurities. The second weight loss was due to the rupture of microspheres, and the third and final weight loss corresponds to almost complete decomposition of the drug-loaded silica microspheres due to pyrolysis [29]. The highest weight loss analyzed in the case of DLMs exposed a higher loading of drugs in the DLMs matrix.

### 2.7. X-ray Diffraction Analysis (XRD)

XRD was performed to investigate the degree of crystallinity of the drugs entrapped into the formulation. The XRD patterns were obtained for LD, NFZ and the optimized drug-loaded microspheres. Peaks of LD were observed at 6.9°, 14.2°, 20.8°, 25.2°and 27.8° at a diffraction angle 2θ which showed its high degree of crystalline nature as shown in Figure 4. Characteristic peaks of NFZ were found at 5.1°, 14.3°, 18°, 19°, 22.2°and 24.8° at a diffraction angle 2θ linked to its crystalline nature. The characteristic peaks of silica were found at 22° of diffraction angle 2θ in microspheres as shown in Figure 4 [30]. The absence of peaks of LD and NFZ in the drug-loaded microspheres confirmed that both drugs were fully entrapped in microspheres and is present in amorphous state.

### 2.8. In Vitro Antibacterial Performance

**Figure 5:**
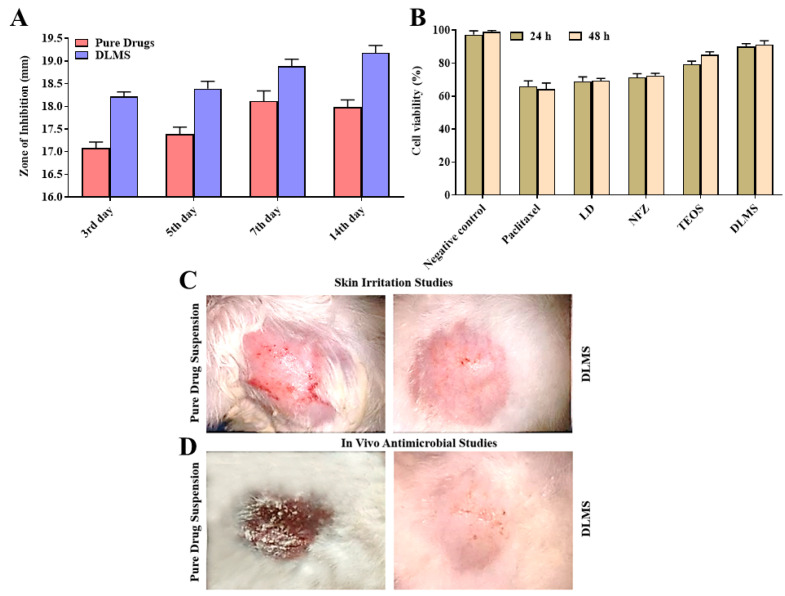
In vitro antibacterial studies demonstrating comparative zone of inhibition (**A**); cytotoxicity studies of NFZ, LD and DLMs (**B**); skin texture images of rabbits obtained at day 14 during skin irritation studies (**C**) [in vivo antimicrobial studies (**D**) for the pure drug suspension treated group and drug-loaded silica microspheres. The values are mean ± SE of three independent experiments.

In vitro antibacterial activities of pure NFZ, LD and NFZ–LD-loaded silica microspheres were investigated on days 3, 5, 7, and 14, and the difference in the zone of inhibitions against *Staphylococcus aureus* species was observed. In this study, *S. aureus* was selected as a test microbe because of its presence and growth at burn wound sites, and its treatment remains a challenge because of its strong resistance to commonly available antibiotics. Both DLMs and drug suspension showed promising antibacterial activity against *S. aureus*, but the zone of inhibition for DLMs was greater than that for drug suspensions as presented in Table 3 and Figure 5, thus showing better antibacterial activity. The suspension of a pure drug combination showed a zone of inhibition of 17.1 mm on day three, while drug-loaded silica microspheres exhibited a zone of inhibition of 18.2 mm. The observations on 5th day depicted that zone of inhibition was 17.4 mm for pure drug suspension and 18.41 mm for drug-loaded microspheres. On day seven, the zone of inhibition increased by 17.8 mm for pure drugs and 18.9 mm for DLMs. For pure drug combinations, the greatest zone of inhibition was 18.00 mm, and for drug-loaded microspheres, it was 19.2 mm. In vitro antibacterial investigations clearly indicated that the NFZ–LD-loaded microspheres had superior action to pure NFZ and LD suspension. The improved antibacterial activity of drug-loaded microspheres may be linked to silica’s antibacterial properties [31]. The availability of drugs from DLMs as compared to simple suspension of drugs was expected to be slow, but the antibacterial activity of DLMs was much higher, which might be due to the presence of silica. The studies of formulations consisting of silica particles loaded with antibiotics are highly encouraging, and such materials were found to exhibit effective antibacterial activity both in vivo and in vitro environments. It is quite evident that the addition of silica increases the antibacterial activity of a drug to a certain limit. Rosemary et al. compared the antibacterial efficacy of ciprofloxacin-loaded silica and free ciprofloxacin solutions by performing a bacteriostatic experiment using the agar dilution method [32]. The newly proposed silica particles loaded with ciprofloxacin showed higher antibacterial activity compared to the free antibacterial substance (ciprofloxacin) against *E. coli*. The presence of silica in a drug delivery system mainly increases the capability of a drug to penetrate well into the bacterial cells that leads to improved antibacterial performance.

### 2.9. Cytotoxicity Studies

Cytotoxicity is one of the most important indicators for the biological evaluation of a formulation and formulation components in in vitro studies. Cytotoxicity studies were assessed using the MTT assay. The principle of the assay is based on the ability of healthy cells to reduce MTT to the purple-colored formazan, while unhealthy/dead cells cannot. The cytotoxic potential of DLMs against breast cancer cell lines (MCF-7) was studied. MCF-7 cells were incubated with 15 µM NFZ and 7.5 mM LD in formulated (DLMs) and free form and TEOs for 24 h and 48 h. After 24 h, about 68% of the cells were viable for NFZ and LD. About 93% of the MCF-7 cells showed viability for DLMs, as shown in Figure 5. After 48 h, cytotoxicity was again evaluated and no significant difference in cell viability of the formulations (DLMs and TEOs) was found compared to 24 h later. The cell viability of TEOs and DLMs was 89% and 92%, respectively. Our findings revealed that DLMs were a more acceptable formulation with minor cytotoxicity compared to NFZ and LD [33].

### 2.10. Skin Irritation Studies

The silica microspheres were prepared with the intention to decreasing the unwanted and uninterrupted interaction of drugs with stratum corneum so that the clinical utility of drugs could be enhanced. The current study demonstrated that the NFZ- and LD-optimized-silica microspheres produced prominently less skin itching compared to pure drugs. The irritable impact of pure NFZ and LD suspension was continuously raised seven after day 14, while the group treated with DLMs showed no sign of skin irritation (Figure 5). The optimized silica DLMs showed an astonishing advantage of enhanced acceptability for skin along with the administration of combination drugs which would bring a rapid eradication of infections [34].

### 2.11. In Vivo Antibacterial Studies

The in vivo efficacy of optimized formulation of NFZ- and LD-loaded silica microspheres was analyzed in rabbit’s model (1.5–2 kg). The studies demonstrated that NFZ- and LD-loaded microspheres contained the greater efficacy of infection eradication because none of the animals had shown a positive culture test, whereas three animals out of six represented positive culture tests in the pure drug suspension group. In the control group, all animals had exhibited a positive culture test (Table 4 and Figure 5). Moreover, the recurrence or regrowth of bacteria was not observed in wounds treated with either the suspension of drugs or the drug-loaded microparticles. The greater occlusive character, enhanced bio adhesiveness and better oxygen supply to the cells because of the super-porous nature of the silica microspheres were responsible for the distinguished effectiveness of the studied drug-loaded silica microspheres. Moreover, there was a complete visible absence of bacterial growth under the applied drug-loaded silica microspheres due to the persistent availability of drugs to the wound site over a prolonged period of time. However, the explanation of the exact mechanism responsible for the enhancement of antibacterial activity due to silica is still insufficient. In order to kill bacteria, the drug must be able to cross the cell membrane and enter the bacteria to produce its effect. In the literature, it has been hypothesized that the silica interact with certain proteins present on the surface of bacteria and thus may open some channels on the surface which cause the drug substance to enter smoothly inside bacteria, thereby killing them [35]. Similarly, some researchers simply proposed that the combination of the antibacterial agent with SiO_2_ may have a synergistic effect on killing bacteria [36].

## 3. Materials and Methods

### 3.1. Materials

LD (Gifted sample by A&K Pharmaceutical Faisalabad, Faisalabad, Pakistan) and NFZ were generously gifted by Pharmawise Labs Pvt. Ltd., Lahore, Pakistan. The Ethyl Silicate (mol. Wt. 208.33), 0.08 M NH4OH (mol. Wt 35.05) and Tween 80 (mol. Wt 1310) were purchased from Sigma-Aldrich^®^ Darmstadt, Germany. The vegetable oil (sunflower oil) of Dalda foods Pvt. Ltd. Lahore, Pakistan was used during these studies.

### 3.2. Preparation of Microspheres

The Sol–gel method was used for the preparation of the drug-loaded silica microspheres. Distilled water and freshly prepared 0.1 M HCl were added to ethyl silicate, and the mixture was stirred well at 200 rpm for 5 min on a magnetic stirrer for the formation of sol. The 50 mg of LD and NFZ was separately dissolved in ethanol and dimethyl formamide, respectively. The obtained drug solutions were added simultaneously into the already prepared sol with continuous stirring. This drug-containing sol was cooled at 4 °C and 0.08 M NH_4_OH was added drop-wise to adjust the pH 5.83, and the GT was noted. Then, the sol was added drop-wise into 100 mL of vegetable oil under constant stirring with a yellow line homogenizer at the speed of 1000 rpm until microspheres precipitated at the bottom of the beaker. The formulated microspheres were obtained by centrifugation at 5000 rpm for 40 min and then dried by lyophilization (Marya Pharmaceutical Turnkey, Model 200121, Shanghai, China) for 24 h. Seventeen formulations of drug-loaded silica microspheres were prepared by changing the level of studied variables as suggested by the applied Box–Behnken design [33] as given in Table 5.

### 3.3. Evaluation of Silica Microspheres

#### 3.3.1. Gelation time (GT) Measurement

During the preparation of the microspheres, a drop-wise addition of 0.08 M NH_4_OH into the drugs containing sol turned the sol into a gel with the addition of base. The time taken for conversion of the sol into viscous gel visually was recorded as a GT [1].

#### 3.3.2. In Vitro Release Studies for NFZ–LD-loaded Microspheres

Dissolution apparatus USP type-II (PT-DT7, Pharma Test Germany) was used for the release study of DLMs at 50 rpm rotation. The dissolution media was made up of a freshly prepared solution of phosphate buffer (pH 6.8) and maintained at the temperature 37 ± 0.5 °C. The DLMs were taken in a dialysis tube (16 mm diameter) containing 5 mL of the dissolution medium. This dialysis tube was tied with a thermos-resistant thread and placed in the dissolution media. After different time intervals (0.5, 1, 2, 3, 4, 5, 6 and 8 h), about a 5 mL sample of dissolution media was withdrawn from each vessel to determine drug content, and the withdrawn sample was replaced by adding 5 mL of freshly prepared media in each vessel. The unknown concentration of NFZ and LD in each withdrawn sample was determined by an already established and validated HPLC method after diluting the obtained sample with a mobile phase [3,18]. The release data was also analyzed for understanding the drug release mechanism by applying different kinetic models [18] as given in Supplementary Materials Table S1.

#### 3.3.3. PY of NFZ–LD-loaded Microspheres

The calculation of the PY of microspheres usually indicates the efficiency of the applied formulation process. Therefore, the PY of the NFZ–LD-loaded microspheres was calculated by dividing the total obtained weight of the microspheres with total amounts of all ingredients (both drugs and ethyl silicate) used to synthesize these microspheres and multiplying it by 100 [20].

(5) $$\mathrm{Percent}\;\mathrm{yield}=\frac{\mathrm{amount}\;\mathrm{of}\;\mathrm{dried}\;\mathrm{microparticles}\;\mathrm{recovered}}{\mathrm{amount}\;\mathrm{of}\;\mathrm{drugs}+\mathrm{amount}\;\mathrm{ethyl}\;\mathrm{silicate}}\times 100$$

#### 3.3.4. Fourier Transforms Infrared Spectroscopy (FTIR)

The FTIR spectra were recorded by using instrument Shimadzu^®^, Tokyo, Japan IR prestige-21. The scanning range was maintained as 400–4000 cm^−1^ with hydraulic pressure of 150 kg/cm^2^, and the resolution was kept at 2 cm^−1^. For the FTIR analysis, small discs were prepared by mixing the sample with KBr (IR grade) [37].

#### 3.3.5. Thermal Analysis

For thermal analysis, NFZ, LD and DLMs were exposed to a combined instrument for TGA-DSC (model: SDT Q 600, T.A^®^, Newcastle, DE, USA). The samples (weight: 1 mg) were placed in an aluminum pan and heated at the rate of 20 °C/min from 40 °C–300 °C, while keeping the atmosphere inert by flowing the nitrogen at the rate of 50 mL/min [38].

#### 3.3.6. X-ray Diffraction Analysis (XRD)

The X-ray diffraction analysis was performed for NFZ, LD HCl and DLMs by an advanced D8 X-ray diffractometer (Bruker AXS, Madison, WI, USA)). The samples were irradiated with monochromatized X-rays of Cu-Kα with a voltage of 35 kV and at a current of 40 mA. The XRD patterns were recorded for all samples by scanning samples at a 0.02° (2θ) angle range of 5–70° for 0.5 to 1 s [38].

#### 3.3.7. Scanning Electron Microscope (SEM) Analysis of DLMs

A scanning electron microscope (JEOL/EO^®^ version 1.1 JSM-840, Tokyo, Japan) was used for the determination of the surface morphology of the DLMs. The DLMs were placed on one side of the double adhesive tape in drop form which was then stuck to an aluminum stub, then gold coated to make them conductive under an argon atmosphere. Finally, microphotographs were taken to examine their shape and morphology [39].

#### 3.3.8. Zeta Potential and Size Distribution Analysis for DLMs

The zeta potential and particle size of empty and DLMs were examined by Malvern zeta sizer (Version 7.11 Malvern Instruments Ltd., Malvern, UK). For this purpose, 5 mg of DLMs were dispersed in deionized ultra-pure water containing 0.01% T-80 by minor sonication, and then the glass cuvette was placed in an electrophoretic cell. The size and charge distributions were examined by measuring the electrophoretic mobility of the DLMs at 25 °C in a U-shaped cuvette [40,41].

### 3.4. In Vitro Antibacterial Performance by Diffusion Test

*Staphylococcus aureus* (*S. aureus*) was selected as the bacterial species against which in vitro antibacterial analysis was performed because *S. aureus* is the commonly grown species at the burn wound. The *S. aureus* culture was stored at −80 °C in broth of brain heart which was augmented with 20% glycerol. The diffusion test was performed on a Mueller Hinton agar with slight modification. In different petri plates of Mueller Hinton agar (MHA), the inoculation of *S. aureus* strains was performed with a sterile cotton swab which was presoaked in bacterial suspensions of adjusted strength. The streaking with a cotton swab was completed two times over the whole surface of plates by rotating plates approximately at 60°. Then, a well cavity of 3–4 mm was pierced and 50 μL NFZ–LD-loaded microspheres and a NFZ–LD suspension were introduced into the well cavities of separate plates. These agar plates were placed at room temperature and then incubated under aerobic conditions at 37 °C for 24 h [24,42,43].

### 3.5. Cytotoxicity Studies

In vitro cytotoxicity was carried out by culturing MCF-7 cells (breast cancer cell line) in Dulbecco’s modified Eagle medium (DMEM) with 10% fetal bovine serum (FBS) in 96-well plates [26,42]. MCF-7 cells are more commonly used as in vitro models for drug toxicity as they are well characterized and more easily cultured. The DMEM was continually exchanged after every 48 h. The cells were fed with DMEM without FBS 24 h before performing the cell viability studies. The MCF-7 cells were incubated with 0.5% dispersions of optimized-loaded microspheres (DLMS), NFZ, LD, and TEOS for 24 h and 48 h. Untreated cells were considered as negative control and paclitaxel 2.5 µg/mL (Sigma Aldrich, St. Louis, MO, USA) as positive control. After the completion of incubation, the 3-(4,5-dimethylthiazol-2-yl)-2,5-diphenyltetrazolium bromide (MTT) assay was performed. After removing samples, the cells were washed three times with phosphate-buffered saline (PBS). Then, a working solution of MTT (500 μL) in FBS-free DMEM (0.5 mg/mL) was added to each well, and the cells were incubated for 1 h. Subsequently, the supernatants were removed. The amount of MTT dye reduced to formazan crystals was dissolved in 500 μL dimethyl sulfoxide (DMSO). This solution was then transferred to 1.5 mL tubes and centrifuged at 13,400 rpm for 2 min. The absorbance of the resulting solution was recorded immediately at λ = 570 nm after dilution of the samples with an equal volume of DMSO. Cell viability rates were calculated according to the following equation:

(6) $$\mathrm{Cell}\;\mathrm{viability}\;\left(\%\right)=\frac{A_{s}}{A_{d}}\times \;100$$

Here, *A_d_* is the absorbance measured after treatment with DMEM, and *A_s_* is the absorbance measured after treatment with tested sample dispersions.

### 3.6. Skin Irritation Testing

Topical applied drugs show some side effects such as skin dryness, rashes and itching which ultimately restricts the use of such drugs by patients. The probability of skin irritation of optimized microspheres formulation was compared with a NFZ/LD suspension by performing a Draize patch test on rabbits [27]. The hair from the back of rabbits was removed just 24 h prior to the formulation applications. The optimized DLMs and NFZ and LD suspensions were uniformly applied on the hair-free skin area (3 cm^2^) of different rabbits, and the rabbits were examined for any type of erythema on day 1, day 7 and day 14, and finally an average erythema score was calculated. The erythema score was documented as zero for no sign of erythema, one for minor erythema, two for modest erythema, three for slightly sever erythema and four for severe erythema.

### 3.7. In Vivo Antibacterial Performance

The experiment of in vivo antibacterial and skin irritation was approved by the Ethical Committee of University of Sargodha, Sargodha, Pakistan. For the induction of bacterial infection on rabbits, the strains of S. aureus were used [25]. From the back of rabbits on an area of 3 × 3 cm^2^, the hair was shaved, and the shaved skin area was further scratched by using sandpaper the next day. The prepared inoculum of *S. aureus* (600 mg) was applied on the created hair-free area by using a glass rod. Twelve rabbits were accurately categorized into two groups with six rabbits in each.

Group I: treated with suspension of NFZ/LD

Group II: treated with NFZ/LD-loaded microspheres

After 24 h of infection, the formulations of NFZ–LD-loaded microspheres and pure NFZ/LD suspensions were then applied on the infection sites of the rabbits of two treated groups for fourteen days. The animals were continuously observed visually to detect any alteration in infected skin area after therapy initiation. The antibacterial activity of the two treated groups was comparatively analyzed with respect to treatment time and change in skin texture. From the treated site, the skin scratches were obtained after 14 days of therapy initiation and homogenized with saline (4 mL) by using the tissue homogenizer. A fraction of homogenate was then spotted on MHA medium, and all plates were then incubated at 37 °C for 3 days. In each plate, the numbers of colony forming units (CFUs) were counted to design the logarithm of the number of CFUs for each infected site. The observation of more than one colony of bacteria made the rabbits considered bacteria positive.

## 4. Conclusions

In the current study, controlled release NFZ- and LD-loaded silica microspheres were successfully prepared and optimized by the Box–Behnken design for an effective treatment of skin infections. Morphological and zeta potential studies confirmed smooth surface spherical-shaped-free-flowing silica microspheres having a highly porous structure with a size distribution of 10–100µm and a zeta potential of −28 mV. Both drugs were successfully incorporated in the microspheres without facing any significant chemical or molecular interaction between the NFZ, LD and silica. The incorporation of drugs into silica microspheres resulted in controlled and prolonged delivery of both medicaments at the wound site, causing rapid recovery from infectious wounds. In conclusion, the obtained results of in vitro and in vivo antibacterial studies have clearly demonstrated that the combination therapy administered in a controlled manner seems to be more effective for acute and chronic infectious wounds and can be regarded comparatively safe as compared to conventional modes of drug administration. However, in future, these studies may require some additional experimentation performed on a large population by encapsulating the same or some different antibiotics in silica microspheres to clinically prove the therapeutic efficacy, toxicology and ADRs of the developed novel formulation.

## Figures and Tables

**Table 1:** Results of ANOVA indicating the effect of formulation variables on studied responses with their *p*-value.

Independent Variable	GT (Y_1_)		PY (Y_2_)		NFZ Release (Y_3_)		LD Release (Y_4_)	
Source	F-Value	*p*-Value	F-Value	*p*-Value	F-Value	*p*-Value	F-Value	*p*-Value
Model	15,569.36	<0.0001	105.38	<0.0001	215.24	<0.0001	6.42	0.0114
*X*_1_	1.36 × 10^5^	<0.0001	0.8872	0.3776	1813.57	<0.0001	31.49	0.0008
*X*_2_	0.6027	0.463	185.12	<0.0001	1.27	0.2965	2.69	0.1451
*X*_3_	86.79	<0.0001	525.92	<0.0001	0.0019	0.9662	0.2045	0.6648
*X*_1_ *X*_2_	60.3	0.0001	1.08	0.3323	1.66	0.2388	6.43	0.0389
*X*_1_ *X*_3_	5.43	0.0526	0	0.9964	10.97	0.0129	0.0245	0.88
*X*_2_ *X*_3_	15.37	0.0057	3.71	0.0955	0.0402	0.8468	0.0222	0.8857
*X*_1_^2^	14.9	0.0062	3.08	0.1226	27.46	0.0012	6.02	0.0439
*X*_2_²	1.09	0.3314	21.65	0.0023	19.16	0.0032	4.57	0.07
*X*_3_²	5.79	0.047	7.76	0.0271	0.9374	0.3652	0.41	0.5424

**Table 2:** Composition of optimized formulation with experimental values of prediction error, desirability factor, microsphere size, zeta potential, PY, GT, NFZ and LD release.

Composition of Optimized DLMs		DLMs Responses	Exp. Value	Predicted Value	PE	DF	Size (µm)	ZP (mv)
pH	6.9	GT	92.5	95	2.88	0.921	50 ± 4.65	−28 ± 3.32
Stirring time (min)	150	PY	88.5	91.00	4.04	0.947	-	-
Vegetable Oil concentration (mL)	100	NFZ release	31.5	28.00	4.46	0.892	-	-
		LD release	30.6	25.00	4.46	0.887	-	-

DF: desirability factor; PE: prediction error; Exp.: experimental; ZP: zeta potential.

**Table 3:** A comparison of in vitro antibacterial analysis of DLMs and pure drugs.

Formulation/Treatment	Size of Inhibition Zone (mm)			
	3rd Day	5th Day	7th Day	14th Day
NFZ- and LD-loaded DLMs	18.2	18.41	18.9	19.2
Pure NFZ and LD suspension.	17.1	17.4	17.8	18.00

**Table 4:** Average erythema score for formulations and comparative in vivo antibacterial results.

Sr. No.	Treated Group with Formulation	Average Erythema Scores			In Vivo Antibacterial Performance	
		1st Day	7th Day	14th Day	Rabbits Having Positive Test/Total No of Rabbits	Infected Sites/Log CFU
1	Group I (Control Group)	0	0	0	6/6	6.73 ± 1.67
2	Group II (NFZ/LD suspension)	1	2	4	3/6	3.61 ± 1.21
3	Group III (NFZ–LD-loaded Microspheres)	0	1	0	0/6	0

**Table 5:** Composition of drug-loaded microspheres as designed by the Box–Behnken design and the obtained outcomes of dependent variables.

Formulation (Coded Levels of Ingredients)	Actual Values of Formulation Ingredients			Results of Responses			
	*X*_1_ pH	*X*_2_ Stirring Time (Hours)	*X*_3_ Volume of Oil (mL)	GT (min)	PY (%)	NFZ Release (%)	LD Release (%)
F1 (+1, 0, −1)	8.4	02	50	147 ± 1.23	49 ± 2.32	42 ± 2.54	43 ± 2.71
F2 (−1, 0, 0)	5.8	02	50	09 ± 2.11	48 ± 3.27	31 ± 2.37	33 ± 1.83
F3 (0, 0, 0)	7.2	02	75	76 ± 2.75	58 ± 1.89	35 ± 2.85	37 ± 3.54
F4 (0, −1, +1)	7.2	01	100	72 ± 3.12	62 ± 2.67	36 ± 3.29	38 ± 3.44
F5 (0, +1, −1)	7.2	03	50	77 ± 2.54	48 ± 2.54	37 ± 1.92	38 ± 2.56
F6 (−1, 0, 0)	5.8	02	75	07 ± 1.25	61 ± 3.71	31 ± 3.41	33 ± 2.37
F7 (0, 0, 0)	7.2	02	75	76 ± 3.87	58 ± 3.39	35 ± 3.73	37 ± 1.84
F8 (0, 0, 0)	7.2	02	75	76 ± 3.98	58 ± 3.83	35 ± 2.61	37 ± 3.52
F9 (+1, −1, 0)	8.4	01	75	149 ± 2.07	49 ± 2.59	43 ± 2.87	45 ± 3.42
F10 (0, 0, 0)	7.2	02	75	76 ± 2.92	58 ± 1.97	35 ± 3.14	37 ± 2.31
F11 (−1, −1, 0)	5.8	01	75	06 ± 1.03	44 ± 3.26	31 ± 1.72	32 ± 2.36
F12 (+1, 0, +1)	8.4	02	100	145 ± 3.41	76 ± 2.23	48 ± 1.77	45 ± 3.57
F13 (0, +1, +1)	7.2	03	100	75 ± 2.34	84 ± 3.39	36 ± 2.82	38 ± 3.69
F14 (−1, +1, +1)	5.8	03	100	08 ± 2.32	82 ± 4.17	30 ± 3.81	33 ± 2.81
F15 (0, −1, −1)	7.2	01	50	78 ± 3.52	34 ± 3.81	37 ± 2.89	38 ± 2.56
F16 (0, 0, 0)	7.2	02	75	76 ± 4.18	58 ± 2.96	35 ± 3.15	37 ± 1.83
F17 (+1, +1, 0)	8.4	03	75	144 ± 3.47	64 ± 3.62	44 ± 2.29	45 ± 2.92

## Data Availability

Not applicable.

## Supplementary Materials

The following are available online at https://www.mdpi.com/article/10.3390/molecules27082532/s1, Table S1: Drugs release kinetics models and their mathematical expressions, Table S2: Selection of optimized DLMs [19,20,21,22,34,39].

## Sample Availability

Samples of the compounds DLMs of NFZ and LD are available from the authors.
