# Supplementary material for: Antibacterial and Wound-Healing Activities of Statistically Optimized Nitrofurazone- and Lidocaine-Loaded Silica Microspheres by the Box–Behnken Design
Source: Molecules. 2022 Apr 14;27(8):2532. doi: 10.3390/molecules27082532 (PMC9032706; doi:10.3390/molecules27082532)
Supplement: Supplementary file 1 [file molecules-27-02532-s001.zip › molecules-1661223-supplementary.pdf]

SUPPLYMENTARY DATA

METHODOLOGY

Selection of optimized DLMs

After detailed analysis of twenty formulations of DLMs on the basis of gelation time, PY and drugs release, the numerical optimization technique was used in order to attain the desirable level of PY and drugs release by generating optimal conditions for formulation synthesis. In this technique, the optimized formulation conditions were selected on the basis of having maximum percentage yield but highly controlled release of both drugs alongwith performance of a detailed feasibility investigation. The studied responses of DLMs were also analyzed on the desirability scale (0-1) by using Design expert and the DLMs with desirability factor close to one was considered as optimized [1]. The software suggested optimized DLMs were then formulated, evaluated for further *in vivo*/*in vitro* antibacterial studies, cytotoxicity, skin irritation and wound healing studies. For the optimized DLMs, the comparative analysis of predicted and experimental results of dependent variables was performed to calculate the prediction error (PE) as given in equation 5.

$$\text{PE }(\%) = [(\text{Experimental value} - \text{Predicted value})/\text{Predicted value}] \times 100 \quad (1)$$

Entrapment efficiency of DLMs

For the entrapment efficiency (EE), 25mg of DLMs were weighed accurately, crushed and then added into the 25ml of phosphate buffered saline (pH 7.4). The mixture was stirred (gently) for 24 hours. Sonication was of obtained solution was performed for 15 minutes so that the entrapped drugs completely came out of microspheres. The mixture was filtered carefully, and after suitable dilutions, the drug contents were determined by HPLC method [2-3]. The EE was determined by the following equation;

$$\text{Entrapment efficiency} = \frac{\text{Estimated drug content}}{\text{Theoratical drug content}} \times 100 \quad (2)$$

Drug release kinetic models for NFZ-LD loaded microspheres

The release mechanisms of drugs were studied by the application of five different kinetic models. Mathematical expression of all these applied models are given below in table S1[4];

Table S1. Drugs release kinetics models and their mathematical expressions

**Model**	**Mathematical expression of Model**
Zero order model	$C=K_o t$
First order model	Log C= Log C_o + Kt/2.303 *where C^o is the initial conc.*
Higuchi Model	$Q=Kt^{1/2}$ where *t is time and k is Higuchi rate constant*
Hixon-crowell Model	$C_o^{1/3} - C_t^{1/3} = k_s t$ *where k_s is constant for surface volume and C_t is remaining amount of drug*
Korsmyer-peppas model	$Mt/M\alpha = kt^n$ *where t is time and n is release kinetics constant used to describe the mechanism of drug transport. There are four types of mechanisms namely, Fickian-diffusion ("n" value 0.45), Anomalous also known as non-Fickian diffusion ("n" value 0.45<n<0.89), Case IIA transport ("n" value 0.89) and Super case IIA transport ("n" value higher than 0.89).*

Micromeritics Analysis

Micromeritics deals with the flow behaviour of a formulation and these can be performed by means of different formula as discussed below;

$$I = \frac{Vb - Vt}{Vt \times 100} \tag{3}$$

After calculating tapped volume (V_t) and bulk volume (V_b), the Carr's index (I) was calculated as shown in equation 6. The value of carr's index ranging from 12 to 19% indicates good flow character and a value greater than 21% represents poor flow behaviour [5]. Similarly, hausner's ratio was calculated from bulk density (ρb) and tapped density (ρt) of a formulation as given in following equation 7;

$$\text{Hausner ratio} = \frac{\rho t}{\rho b} \tag{4}$$

The value of ratio lower than 1.25 indicates good flow character while its value greater than 1.25 shows poor flow properties [5]. Another micromeritics parameter was the angle of repose which was calculated by passing a specified amount of DLMs through the funnel on a plain paper sheet.

From the heap of DLMs, the radius (r) and height (h) were measured and the angle of repose was calculated by using following equation;

$$\tan\theta = \frac{h}{r} \quad (5)$$

The angle of repose less than 30° usually confirmed a free flow parameter of a formulation.

RESULTS AND DISCUSSION

Selection of optimized formulation

The attainment of optimized silica microspheres formulation was established by numerical optimization process for achieving higher percentage yield (90%) and highly controlled and slow drugs release (25%) at eight hours [1]. Regarding formulation's conditions, the the desirable results for suggested options were prioritized on the basis of desirability factor. The software created optimized DLMs having zeta potential of -28 mV, an average size of 50µm with upgraded PY and prominent controlled drugs release were synthesized (Table 3) which were further examined for their biomedical applications. The desirability factor for all studied variables of DLMs was observed as close to one and the PE was also found to be <05% signifying that the success and adequacy of optimization process.

Entrapment efficiency (EE) of drugs loaded microspheres

The EE helps to determine how much amount of drug is entrapped in silica microspheres for therapeutic purposes. The entrapment efficiency of NFZ and LD has been observed in the range of 65.3% to 71.9% and 66.2% and 72.7% respectively. The formulation F14 has showed maximum EE of 71.9% for NFZ and 72.7% for LD. It has been seen that there is no profound change in entrapment efficiency because for silica microspheres EE depends upon the change in drug concentration. In microspheres, the concentration of NFZ and LD have been remained constant in all seventeen formulations [6].

Micromeritics Analysis

The drugs loaded silica microspheres must have suitable flow behavior if they are supposed to be packed in capsule, transdermal patch, tablet or any other dosage form which would further ensure the appropriate administration of DLMs to patients. The results of analysis for all formulations are presented in Table S2. The studied parameters such as stirring time, oil concentration and pH have produced significant impact on DLMs. Mild acidic conditions and higher stirring time had shown positive effect on flow character of DLMs. The car's index for all DLMs formulations ranging from 9 to 18 suggests an excellent flow character. For most of the

formulations, the angle of repose also found to be <20° verifying the better flow behaviour DLMs. In the same way, the findings of hausner's ratio had also established the good micromeritics of DLMs because it was observed to be <1.5 for all formulations.

Table S2. Zeta potential, microsphere size, polydispersity Index and micromeritics analysis of all DLMs

Formulations	Hausner's ratio	Carr's index	Angle of repose	Size (µm)	Zeta Potential (mv)	PDI
F1	1.13±2.67	15±2.69	18±3.59	65±3.79	29±3.46	0.611
F2	1.12±2.82	14±1.68	17±2.52	65±5.79	28±2.37	0.603
F3	1.15±2.59	13±2.68	17±2.72	50±4.19	27±1.18	0.552
F4	1.14±2.12	13±2.64	18±2.81	59±6.68	28±4.26	0.593
F5	1.13±2.78	11±1.78	18±2.98	66±6.81	27±3.21	0.613
F6	1.12±1.71	13±1.58	19±3.38	65±3.95	26±5.63	0.532
F7	1.14±2.32	13±3.33	17±2.54	50±4.19	27±1.18	0.551
F8	1.13±2.39	13±2.29	17±3.28	50±4.19	27±1.18	0.551
F9	1.12±3.12	15±1.84	17±2.81	63±4.98	28±4.67	0.621
F10	1.14±2.93	13±2.45	17±2.17	50±4.19	27±1.18	0.55
F11	1.12±1.97	15±1.69	17±1.88	68±4.94	28±3.84	0.591
F12	1.11±2.51	12±2.92	17±3.61	60±3.23	27±2.44	0.511
F13	1.02±1.36	10±3.87	14±3.43	51±3.39	28±3.81	0.493
F14	1.12±1.53	11±2.69	16±2.78	56±4.73	27±3.19	0.501
F15	1.14±2.12	14±1.71	18±3.44	70±6.68	27±4.52	0.612
F16	1.13±2.67	13±2.82	17±2.31	50±4.19	27±1.18	0.551
F17	1.12±3.82	13±1.93	17±2.49	61±5.74	26±3.29	0.562

REFERENCES

1. Matlhola K, Katata-Seru L, Tshweu L, Bahadur I, Makgatho G, Balogun M. Formulation and optimization of Eudragit RS PO-tenofovir nanocarriers using Box-Behnken experimental design. J. Nanomater. 2015; Article ID 630690.

2. Shaalan AR, Belal ST. HPLC-DAD stability indicating determination of Nitrofurazone and Lidocaine Hydrochloride in their combined topical dosage form. J. Chromatogr Sci. 2010;48:647-53.

3. Barakat NS, Yassin AE. In vitro characterization of carbamazepine-loaded precifac lipospheres. Drug Deliv. 2006;13(2):95-104.

4. Dash S, Murthy PN, Nath L, Chowdhury P. Kinetic modeling on drug release from controlled drug delivery systems. Acta Pol Pharm. 2010;67(3):217-23.
5. Jelvehgari M, Atapour F, Nokhodchi A. Micromeritics and release behaviours of cellulose acetate butyrate microspheres containing theophylline prepared by emulsion solvent evaporation and emulsion non-solvent addition method. Arch Pharm Res. 2009;32(7):1019-28.
6. Yilmaz E, Bengisu M. Drug entrapment in silica microspheres through a single step sol–gel process and in vitro release behavior. J Biomed Mater Res Part B: Appl Biomater. 2006; 77(1):149-55.
